# A diagnostic model based on ^18^F-FDG PET/CT parameters in improving the differential diagnosis of invasive thymic epithelial tumors and anterior mediastinal lymphomas

**DOI:** 10.1186/s12880-023-01185-1

**Published:** 2024-01-08

**Authors:** Shuo Zhou, Pokwan Tsui, Meifu Lin, Guobao Chen, Wenxin Chen, Xiangran Cai

**Affiliations:** 1 Department of Nuclear Medicine, Shengli Clinical Medical College of Fujian Medical University, Fujian Provincial Hospital, Fuzhou City, Fujian Province China; 2 Medical Imaging Center, First Affiliated Hospital of Jinan University, No. 613 West Huangpu Avenue, Tianhe District, Guangzhou, 510630 Guangdong China

**Keywords:** Invasive TETs, Anterior mediastinal lymphomas, ^18^F-FDG PET/CT, Differential diagnosis

## Abstract

**Background:**

Accurately distinguishing between invasive thymic epithelial tumors (TETs) and anterior mediastinal lymphoma before surgery is crucial for subsequent treatment choices. But currently, the diagnosis of invasive TET is sometimes difficult to distinguish from anterior mediastinal lymphoma.

**Objective:**

To assess the application of fluorine-18-fluorodeoxyglucose (^18^F-FDG) positron emission tomography/computer tomography (PET/CT) in the differential diagnosis of TETs and anterior mediastinal lymphomas.

**Methods:**

^18^F-FDG PET/CT images of 133 invasive TETs and anterior mediastinal lymphomas patients were retrospectively analyzed. In particular, the tumor’s longest diameter and maximum standardized uptake value (SUVmax) were evaluated. The SUVmax and longest diameter values of the two groups were analyzed by using the receiver operating characteristic (ROC) curve to determine the optimal threshold and diagnostic efficiency.

**Results:**

Age, myasthenia gravis, SUVmax and tumor longest diameter differed significantly between invasive TETs and anterior mediastinal lymphomas patients. The tumor location, calcification, relationship with adjacent vessels and distant metastasis differed significantly between the groups. The ROC analysis showed an AUC for SUVmax and tumor longest diameter of 0.841 and 0.737. Respectively, the cutoff values with the best diagnostic performance were 9.65 (sensitivity: 77.78%, specificity: 81.97%) and 6.65 (sensitivity: 80.56%, specificity: 62.30%) for SUVmax and tumor longest diameter. The diagnostic model of SUVmax, calcification, relationship with surrounding blood vessels, lymph node metastasis and lung metastasis in the highest AUC of 0.935 (sensitivity: 90.16%, specificity: 88.89%). In addition, we incorporated splenic involvement and metastatic sub-diaphragmatic lymph node into Model 2 as a new predictive model 3 for differential diagnosis and found a significant improvement in the diagnostic performance of Model 3.

**Conclusion:**

The diagnostic model composed of ^18^F-FDG PET parameters is improving the differential diagnosis of invasive TETs and anterior mediastinal lymphomas.

## Introduction

Thymic epithelial tumors (TETs), including thymomas and thymic carcinomas and thymic neuroendocrine tumors are the most common malignancies in the anterior mediastinum in adults [1]. The 2004 version of the World Health Organization (WHO) classification subdivides TETs into A, AB, B1, B2, B3 and C (thymic carcinomas) [2]. Of note, most types A and AB thymomas have low malignant potential, types B1, B2 and B3 are more aggressive, and type C is a highly aggressive tumor [3]. Types A and B thymomas are usually at stage I-II while most type B2/B3/C thymomas are at stage III-IV [4]. On the other hand, Thymic lymphomas is one of the most common malignant lymphomas originating in the anterior mediastinal region, which is hard to differentially diagnosis with TETs [5]. Practically, optimal therapeutic options are different for TETs and anterior mediastinal lymphomas. Surgery is the mainstay treatment for TETs, while radiotherapy and chemotherapy are more preferred for anterior mediastinal lymphomas [6, 7]. Thus, making an accurate differential diagnosis between TETs and anterior mediastinal lymphomas is very critical to avert thoracotomy for anterior mediastinal lymphomas patients [8, 9].

Currently, magnetic resonance (MR) and computed tomography (CT) are widely used to evaluate anterior mediastinal masses, but there are still some deficiencies in distinguishing histological subtypes [10, 11]. Different from invasive TETs and anterior mediastinal lymphomas, most of noninvasive TETs can be provided definitive diagnosis by using MR or CT scan [12]. Clinically, there is considerable overlap between invasive TETs and anterior mediastinal lymphomas in imaging features [13], differential diagnosis remains a puzzle.

Fluorine-18-fluorodeoxyglucose (^18^F-FDG) positron emission tomography/CT (PET/CT) is now mainly used for diagnosis, staging, detection of recurrence and response evaluation of many malignant tumors [14, 15]. Several studies have confirmed the value of ^18^F-FDG PET/CT in the differentiation of benign and malignant anterior mediastinal masses [16, 17]. Furthermore, some studies also have found that ^18^F-FDG PET/CT has excellent application value in the differentiating thymoma and anterior mediastinal lymphomas [18, 19]. Most studies have focused on differentiating thymoma and anterior mediastinal lymphomas while ignoring the different degree of malignancy among thymoma subtypes. In fact, the diagnosis of invasive TETs is sometimes difficult to distinguish from anterior mediastinal lymphomas [20]. To our knowledge, there have been relatively few studies on the comparisons between invasive TETs and anterior mediastinal lymphomas. Therefore, we expect that ^18^F-FDG PET/CT image features combines with clinical information can play a critical role in differentiating invasive TETs and anterior mediastinal lymphomas.

In this study, we retrospectively investigated the differences of invasive TETs and anterior mediastinal lymphomas and to assess whether ^18^F-FDG PET/CT image features combines with clinical information can distinguish invasive TETs and anterior mediastinal lymphomas. Furthermore, we constructed the diagnostic model composed of ^18^F-FDG PET parameters and evaluated the diagnosis efficiency of this model.

## Patients and methods

### Ethical approval

The study conforms to the ethical guidelines in accordance with the Helsinki Declaration as revised in 2013 and was approved by Fujian Provincial Hospital review board and ethical committee.

### Patients

**Fig. 1:**
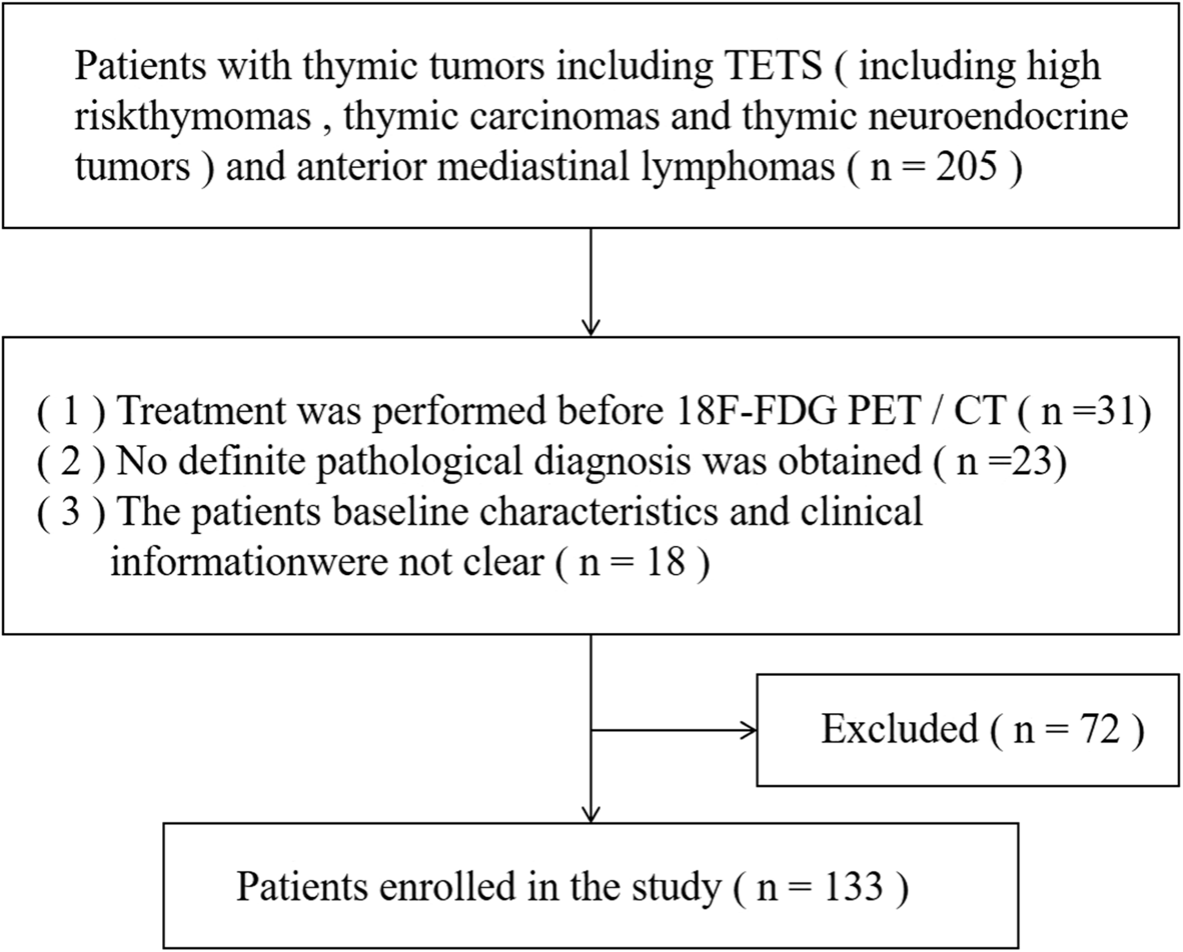
Patient inclusion flowchart

Between January 2013 and January 2018, a total of 205 patients with thymic tumors including invasive TETs and anterior mediastinal lymphoma were retrospectively recruited. All patients were required to meet all the following inclusion criteria: (1) The diagnosis of invasive TETs or anterior mediastinal lymphoma were confirmed by surgical pathology or needle biopsy; (2) ^18^F-FDG PET/CT was performed before treatment; (3) clinical information of patients was complete. Finally, 133 patients were included in the analysis (Fig. 1). The clinical information of all patients was summarized in Table 1.

**Table 1:** Clinical and ^18^F-FDG PET/CT image parameter comparisons between invasion thymoma and anterior mediatinal lymphoma patients

Histopathologic Diagnosis	Invasive TETs (*n* = 61, mean $\varvec{\pm }$ SD)	Anterior mediatinal lymphomas (*n* = 72, mean $\varvec{\pm }$ SD)	$\varvec{X}^{\varvec{2}}\varvec{/t/Z}$	*p* value
Age (years, mean ± SD)	55.84 ± 11.43	28.97 ± 10.37	-14.20	<0.001
Gender				
*Male*	33	40	0.028	0.866
*Female*	28	32		
Location				
*No offset*	37	65	16.21	<0.001
*Offset*	24	7		
Tumor longest diameter (cm)	6.72 ± 2.99	7.99 ± 1.83	3.02	0.003
SUVmax	7.63 ± 4.58	15.68 ± 8.18	6.83	<0.001
Tumor Border				
*Blurred*	45	54	0.026	0.871
*Sharp*	16	18		
Peripheral Vascular Structure				
Encompass	9	25	6.919	0.009
*Infiltrate*	52	47		
Calcification				
*Yes*	18	0	24.571	<0.001
*No*	43	72		
Distant metastasis				
*Yes*	33	26	5.137	0.023
*No*	27	46		
Metastatic below diaphragm lymph node				
*Yes*	7	19		
*No*	54	53	3.602	0.057
Splenic involvement^a^				
*Yes*	0	21		
*No*	61	51	-	<0.001

*CT* computer tomography, *SUVmax* maximum standardized uptake value

^a^Fisher’s exact test

### Image acquisition protocol

All patients fasted for at least 6 hours before ^18^F-FDG administration. Patients with a blood glucose level higher than 180 mg/dL (10.0 mmol/L) or lower than 72 mg/dL (4.0 mmol/L) were rescheduled. Patients were implanted with indwelling intravenous three-way pipes, followed by ^18^F-FDG manual administration with 5.5 MBq (0.15 mCi) per body weight. PET/CT studies were acquired from mid-thigh to the base of the skull [21] using a total-body PET/CT scanner (Discovery LS, GE Healthcare, Milwaukee, WI.) after 50 $\sim$ 60 min resting in dark room. CT scans (tube current 120 mAs, voltage 140 kV, rotation time 0.5 s, pitch 1.35, and slice thickness 5 mm.) of the torso were obtained which reconstructed in a 512 $\times$ 512 matrix. The thoracic CT images were reconstructed by using a standard kernel. Emission data were acquired with 4 min/bed position in 2-dimensional mode; and PET images were reconstructed using ordered subset expectation maximization (OSEM).

### Image analysis

All images evaluation has been performed in a functional imaging workstation (GE, Xeleris version 3.0) and reviewed in standard planes. Two nuclear physicians with 10 years of FDG PET/CT diagnostic experience and fellowship trained in thoracic imaging independently analyzed the PET/CT images blinded to the clinical data. Discordance was resolved by consensus. Semiquantitative parameter maximal standardized uptake value (SUVmax), metabolic tumor volume (MTV), total lesion glycolysis (TLG) was measured semiautomatically over the volume of interests (VOIs) of metabolically active lesions. Thoracic CT images were reviewed by two board-certified radiologists with more than 10 years of experience blinded to the PET images. CT images indicators include tumor location, edge, adjacency and distant metastasis.

### Statistical analysis

All statistical analysis was conducted using SPSS (version 20.0; IBM, Armonk, New York, USA). Unpaired Student’s t test and Chi-square test (or Fisher’s exact tests) were used to examine differences between the groups. The differences in age, SUVmax and tumor longest diameter values between TETs and anterior mediastinal lymphoma were analyzed using Student’s t test, while that Chi-square test was used for the position, boundary, calcification, relationship with surrounding blood vessels and distant metastasis. Single factor analysis screened out statistically significant analysis indicators (*P *< 0.05) and conducted multivariate binary logistic regression analysis to determine the analysis indicators with independent risk factors (*P *< 0.05) and construct a model. The subject operating characteristic curve (ROC) was plotted for the model and independent risk factors. The optimal diagnostic efficacy parameters [sensitivity (Sen), specificity (Spe), area under curve (AUC), and 95% confidence interval] were determined using the maximum Youden index. Diagnostic model was conducted based on multivariate logistic regression analysis. A ROC curve analysis was used to examine SUVmax and tumor longest diameter using medcalc software (version 20.0.3). All comparisons were two-sided and *P* value less than 0.05 was considered as statistical significance. All values in the text and tables were given as the mean ± SD (standard deviation).

## Results

### Clinical characteristics

In this study, a total of 133 patients were included in the analysis, including 61 invasive TETs and 72 thymic lymphomas patients. As shown in Table 1, our data was indicated that myasthenia gravis (66.7%, 38 of 61 patients vs. 0%, 0 of 72 patients, *P* < 0.001) and age (55.84 ± 11.43 vs. 28.97 ± 10.37, *P* < 0.05) were significantly different between the invasive TETs and anterior mediastinal lymphoma patients. And there was no difference between the two groups with respect to gender (%male-54.1% vs. 55.6%; *P* $=$ 0.866). Also, pathological type of malignancies in the anterior mediastinum was shown in Table 2. Thymic Carcinoma was the most common subtype in the thymic epithelial tumors, and diffuse large B-cell lymphoma is the most in the anterior mediastinal lymphomas.

**Table 2:** Pathological type of malignancies in the anterior mediastinum

Pathological type	Number
Thymic epithelial tumors	61
Thymoma(B2)	14
Thymoma(B3)	19
Thymic Carcinoma	28
Anterior mediastinal lymphomas	72
Hodgkin lymphoma	16
Diffuse large B-cell lymphoma	28
T lymphoblastic lymphoma	11
Follicular lymphoma	7
T-cell lymphoma	10

### Comparison of ^18^F-FDG PET/CT imaging features between invasive TETs and anterior mediastinal lymphomas

**Fig. 2:**
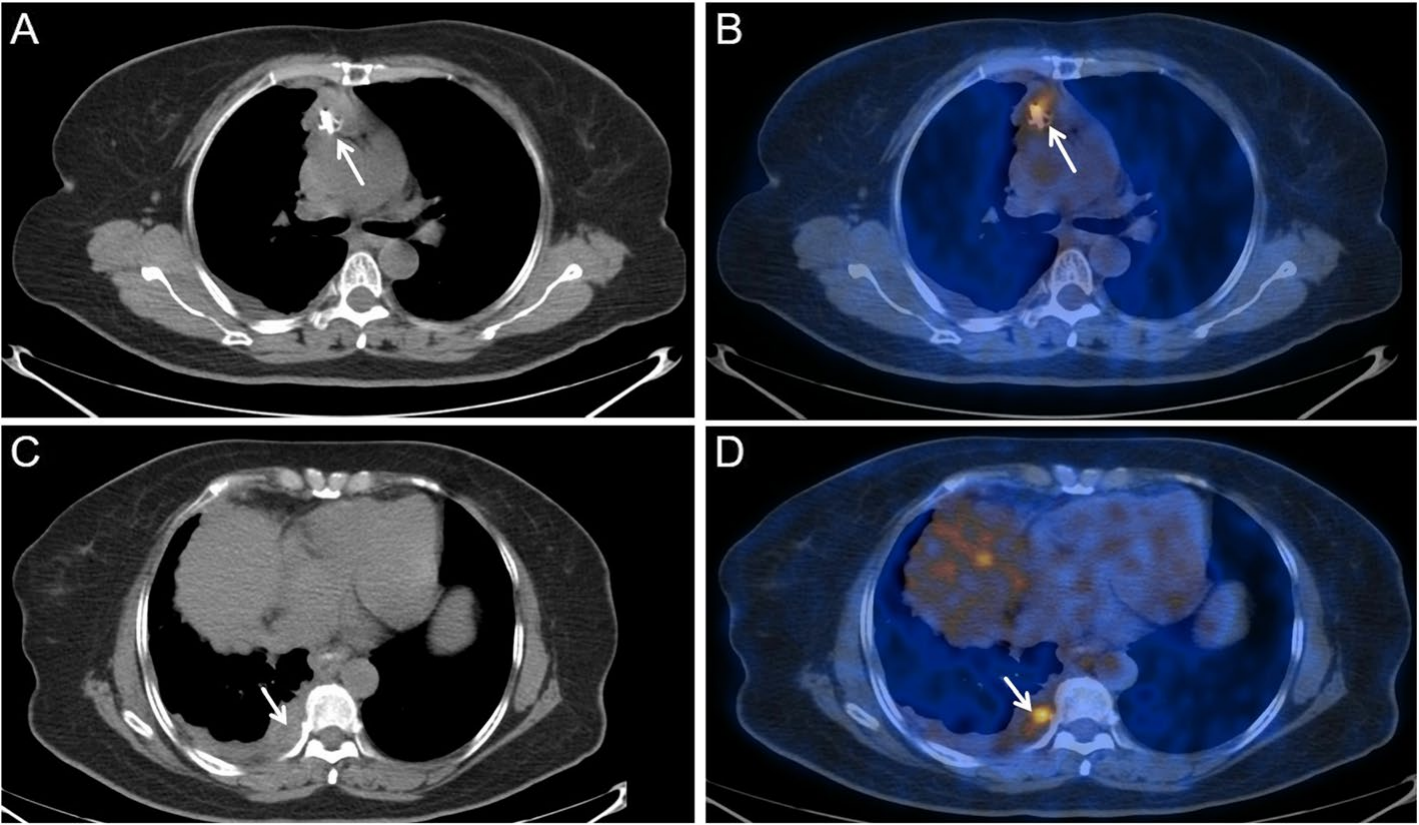
PET/CT findings of a 62-year-old patient with type B3 thymoma. **A** Right anterior medial malleolus soft tissue mass shadow, adjacent blood vessels were not clear and calcified calcification inside the tumor was observed (arrow). **B** A high metabolism signal (arrow) was seen in the tumor, SUVmax $=$ 3.9. **C** A small amount of fluid in the right thoracic cavity (arrow). **D** Right pleural hypermetabolism nodules (arrow), SUVmax $=$ 2.5

**Fig. 3:**
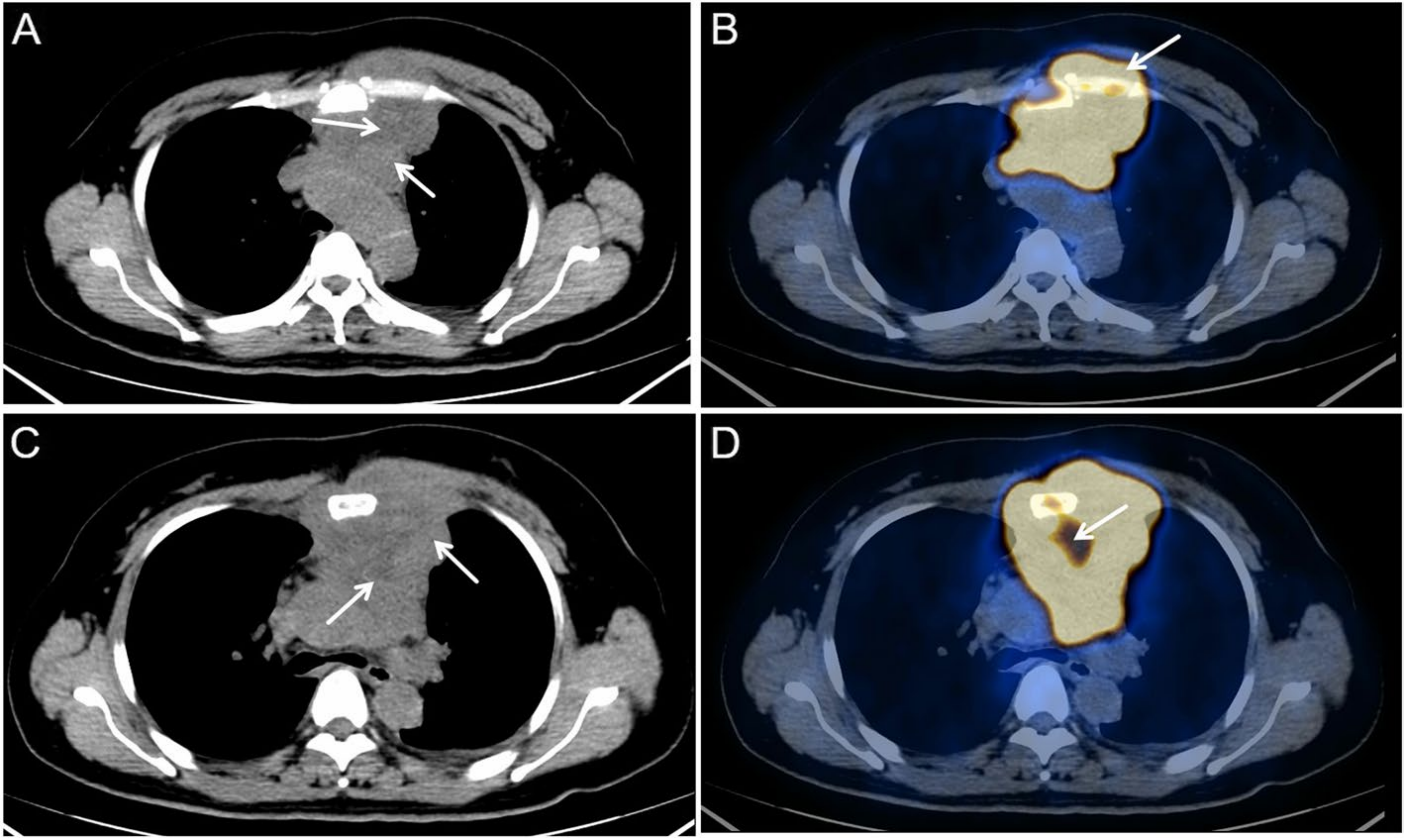
PET/CT findings of a 23-year-old patient with diffuse large B-cell lymphoma. **A** A large soft tissue mass in the anterior medial malleolus which was composed of multiple nodules. Tumor invaded into the front chest wall and partial adjacent large blood vessels (arrow). **B** Significant radioactive abnormal signals (arrow) in the tumor, SUVmax $=$ 35.5. **C** Tumor edge was lobulated with a fissure-like necrotic region inside the tumor (arrow). **D** Radioactive defect inside necrotic region (arrow)

Comparisons of imaging features between invasive TETs and anterior mediastinal lymphomas were shown in Table 1. There were significant differences between invasive TETs and anterior mediastinal lymphomas groups in calcification (29.5%, 18 of 61 patients vs. 0%, 0 of 72 patients, *P* < 0.001), distant metastasis (55.7%, 34 of 61 patients vs. 36.1%, 26 of 72 patients, *P* $=$ 0.023), encased vessels (14.8%, 9 of 61 patients vs. 34.7%, 25 of 72 patients, *P* $=$ 0.009), while no differences in blurred tumor borders (73.8%, 45 of 61 patients vs. 75.0%, 54 of 72 patients, *P* $=$ 0.871). Furthermore, the mean (±SD) values for tumor largest diameter in invasive TETs and anterior mediastinal lymphomas were 6.72± 2.99 and 7.99 ±1.83 cm. The tumor largest diameter for invasive TETs was significantly higher than that in anterior mediastinal lymphomas (*P* $=$ 0.003). The mean (±SD) values for SUVmax in invasive TETs and anterior mediastinal lymphomas were 7.63± 4.58 and 15.68± 8.18 cm. The SUVmax for invasive TETs was significantly lower than that in anterior mediastinal lymphomas (*P* < 0.001). Representative images of invasive TETs and anterior mediastinal lymphomas were shown in Figs. 2 and 3.

### The differentiating efficacy of SUVmax, tumor largest diameter and diagnosis models in invasive TETs and anterior mediastinal lymphomas

**Fig. 4:**
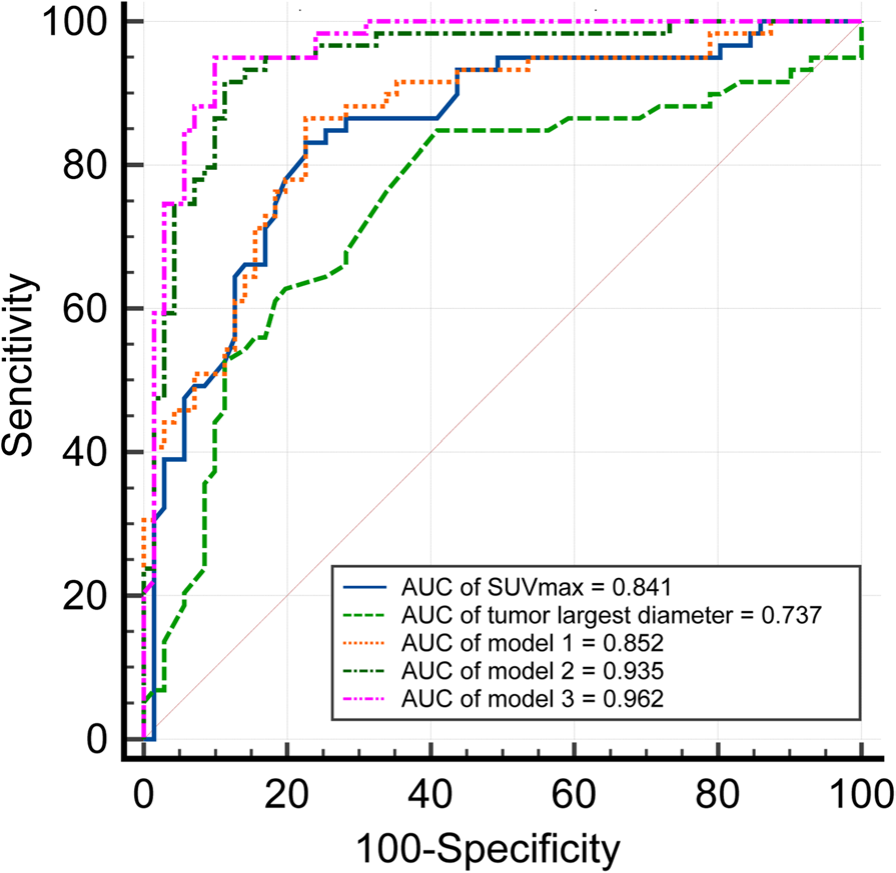
The ROC curves of tumor largest diameter, SUVmax, model 1, model 2 and model 3. ROC analysis to determinate the sensitivity and specificity of the tumor largest diameter, SUVmax, model 1, model 2 and model 3 for differentiating invasive TETs from anterior mediastinal lymphoma

As shown in Fig. 4 and Table 3, ROC curve analysis was used to evaluate the differential efficacy of SUVmax and tumor largest diameter. ROC analysis showed an AUC for SUVmax and tumor largest diameter of 0.841 and 0.737. Respectively, the cutoff values with the best diagnostic performance were 9.65 (sensitivity: 77.78%, specificity: 81.97%) and 6.65 (sensitivity: 80.56%, specificity: 62.30%) for SUVmax and tumor largest diameter. Then, two different diagnostic models including model 1: SUVmax plus tumor largest diameter, model 2: SUVmax, calcification, relationship with surrounding blood vessels, metastasis (lymph node and lung) were constructed by logistic regression analysis. The results showed that the model 2 with SUVmax, calcification, relationship with surrounding blood vessels, metastasis (lymph node and lung) resulted in a highest AUC of 0.935 (sensitivity: 90.16%, specificity: 88.89%) (Fig. 4 and Table 3). The AUC of diagnostic model 2 was better than tumor largest diameter, SUVmax, and model 1 with SUVmax and tumor largest diameter (all *P* < 0.05). In addition, we incorporated splenic involvement and metastatic sub-diaphragmatic lymph node into Model 2 as a new predictive model 3 for differential diagnosis and found a significant improvement in the diagnostic performance of Model 3 (Fig. 4 and Table 3).

**Table 3:** Differential diagnostic efficiency of ^18^F-FDG PET/CT parameters and different diagnosis models between invasive TETs and anterior mediastinal lymphomas

Parameters/model	AUC	Sensitivity	Specificity
Tumor longest diameter	0.737	80.56%	62.30%
SUVmax	0.841	77.78%	81.97%
Model 1	0.852	85.25%	77.78%
Model 2	0.935	90.16%	88.89%
Model 3	0.962	94.92%	90.14%

Model 1: tumor longest diameter plus SUVmax

Model 2: SUVmax plus calcification plus relationship with surrounding blood vessels plus distant metastasis (lymph node and lung)

Model 3: Model 2 plus Metastatic below diaphragm lymph node and splenic involvement

## Discussion

Thymic tumors and lymphomas, while rare, are the most frequent tumors of the anterior mediastinal compartment in adults [22]. Most of non-invasive TETs present as sharply demarcated round or oval soft tissue masses in anterior mediastinal on CT, which usually can be accurately diagnosed [23]. On the contrary, imaging features of CT between invasive TETs and anterior mediastinal lymphoma frequently overlap and sometimes are difficult to differentiate, including lobulated, off-midline and mass effect [23, 24]. Although ^18^F-FDG PET/CT already has been a common clinical diagnostic tool for anterior mediastinal tumors and useful for differentiating benign from malignant tumors, the differential diagnosis between invasive TETs and anterior mediastinal lymphoma is still not well resolved. Importantly, treatment guidelines of invasive TETs and anterior mediastinal lymphomas are distinct. Surgical resection is the mainstay of treatment in invasive TETs, while chemotherapy is more preferred for lymphoma [6, 16, 25]. Accurate diagnosis of invasive TETs and anterior mediastinal lymphomas is critical in planning treatment strategies. However, most studies have focused on the differential diagnosis of thymoma and anterior mediastinal lymphomas while ignoring the different degree of malignancy among thymoma subtypes. In our study, we are primarily focused on comparing the disease characteristics between invasive TETs and anterior mediastinal lymphomas by using ^18^F-FDG PET/CT combined with clinical information.

Mediastinal lymphomas is predominantly occurred in young adult life [25]. Previous studies reported that there was a certain value for the age in differentiating TETs and anterior mediastinal lymphomas [18, 26]. In this current study, the mean age of patients with invasive TETs was significantly higher than that of anterior mediastinal lymphomas patients (55.84 ± 11.43 years vs 28.97 ± 10.37 years, *P* < 0.001). Therefore, the present study supports the previous finding that age is associated with the differential diagnosis of invasive TETs and anterior mediastinal lymphomas. Additionally, myasthenia gravis is an important paraneoplastic manifestation of TETs patients, but may also present in patients with anterior mediastinal lymphomas [27]. Notably, our study reveals that 52.5% (38/61) invasive TETs patients with myasthenia gravis while none of the 72 patients with anterior mediastinal lymphomas had myasthenia gravis. Overall, we demonstrated the differences in age and myasthenia gravis between invasive TETs and anterior mediastinal lymphomas patients.

^18^F-FDG PET/CT metabolic parameters SUVmax is independent variables to predict malignancy [28]. Recent research suggests that there are certain clinical value for SUVmax in differentiating benign and malignant TETs [29]. Giorgio Treglia et al demonstrated that the mean SUVmax in thymic carcinomas was significantly higher than low-risk thymomas [30]. Moreover, SUVmax also can be used in differentiating TETs and anterior mediastinal lymphomas [18, 31]. Catherine T Byrd et al reported that anterior mediastinal tumors with SUVmax less than 7.50 were likely thymoma while anterior mediastinal lymphomas were likely with SUVmax greater than 12.85, and as for tumors with SUVmax between 7.50 and 12.85, it was still need to be biopsied to rule out lymphoma [19]. In fact, most of the anterior mediastinal lymphomas were with high SUVmax value, while invasive TETs with high-grade malignancy are usually with high SUVmax value. Here, our results reveal that the mean SUVmax of patients with anterior mediastinal lymphomas was significantly higher than that of invasive TETs patients (7.63 ± 4.58 vs 15.68 ± 8.18, *P* < 0.001). Meanwhile, we found that the mean maximal diameter of patients with invasive TETs was shorter than that of anterior mediastinal lymphomas patients. (6.72 ± 2.99 cm vs 7.99 ± 1.83 cm, *P* <0.001). ROC analysis found that SUVmax (AUC $=$ 0.840) and tumor largest diameter (AUC $=$ 0.737), suggesting that SUVmax could play an important role in the differentiation of invasive TETs and anterior mediastinal lymphomas. Furthermore, the diagnostic model 2 (SUVmax plus calcification plus relationship with surrounding blood vessels plus lymph node metastasis plus lung metastasis) could significantly improve the diagnostic ability compared to tumor largest diameter, SUVmax and model 1 (SUVmax plus tumor largest diameter).

The presence of lesions in the subdiaphragmatic nodes and spleen is important for distinguishing between different conditions in clinical practice. However, in our analysis, we found that there was no statistically significant association between lesions in the subdiaphragmatic nodes and spleen. Unfortunately, 19.5% of patients in our study had metastatic subdiaphragmatic lymph nodes and 8.3% had infiltration of the spleen. However, when we evaluated the diagnostic performance of these parameters alone, we found that they were not very accurate, with AUC values of 0.574 and 0.646 respectively. Therefore, we incorporated these parameters into a new predictive model (Model 3) for differential diagnosis and found that it significantly improved the diagnostic performance compared to the previous models.

The utilization of diagnostic models can enhance the accuracy of disease diagnosis and aid clinicians in making treatment decisions. By relying on a few key parameters, clinicians can accurately diagnose diseases without solely relying on image interpretation. This can potentially save time and resources, leading to more efficient and effective patient care. This study had some shortcomings that should be acknowledged. Our study was a retrospective cohort study, and the results need to be confirmed in a prospective study. In addition, although this study included many cases, all data were from single-center.

## Conclusion

In conclusion, the diagnostic model composed of ^18^F-FDG PET parameters is improving the differential diagnosis of invasive TETs and anterior mediastinal lymphomas. We also reveal the differences in age and myasthenia gravis between invasive TETs and anterior mediastinal lymphomas patients. ^18^F-FDG PET/CT combined with clinical information present an important diagnostic modality for differential diagnosis of invasive TETs and anterior mediastinal lymphomas, and in turn to provide accurate therapeutic strategies for mediastinal malignancies.

## Data Availability

All relevant data are within the article.
